# Increase in pertussis cases along with high prevalence of two emerging genotypes of *Bordetella pertussis* in Perú, 2012

**DOI:** 10.1186/s12879-016-1700-2

**Published:** 2016-08-17

**Authors:** H. Bailon, N. León-Janampa, C. Padilla, D. Hozbor

**Affiliations:** 1 Laboratorio de Biotecnología y Biología Molecular, Centro Nacional de Salud Pública, Instituto Nacional de Salud, Ministerio de Salud, Lima, Peru; 2 Departamento de Ciencias Biológicas, Laboratorio VacSal del Instituto de Biotecnología y Biología Molecular, Facultad de Ciencias Exactas Universidad Nacional de La Plata, CONICET, La Plata, Argentina

**Keywords:** Pertussis, *Bordetella pertussis*, Genotype, Pulsed-field gel electrophoresis, Perú

## Abstract

**Background:**

As has occurred in many regions worldwide, in 2012 the incidence of pertussis increased in Perú. This epidemiologic situation has been associated with a waning vaccine-induced immunity and the adaptation of *Bordetella pertussis* to vaccine-induced immunity along with improved diagnostic methods.

**Methods:**

The study comprised a total of 840 pertussis-suspected cases reported in Perú during 2012. We summarize here the distribution of pertussis cases according to age and immunization status along with the immunization-coverage rate. Laboratory diagnosis was performed by culture test and real-time polymerase-chain reaction (PCR). *B. pertussis* bacteria recovered from infected patients were characterized by pulsed-field gel electrophoresis (PFGE), and the DNA sequencing of the pertussis-toxin (promoter and subunit A), pertactin, and fimbriae (*fim*2 and *fim*3) genes.

**Results:**

From the total pertussis-suspected cases, 191 (22.7 %) infections were confirmed by real-time PCR and 18 through cultivation of *B. pertussis* (2.1 %), while one infection of *B. parapertussis* (0.11 %) was also detected by culture. Pertussis was significantly higher in patients that had had 0–3 vaccine doses (pentavalent vaccine alone) than in those who had had 4–5 vaccine doses (pentavalent plus DwPT boosters) at 94.3 vs. 5.7 %, respectively (*p* < 0.00001). The relative risk (RR) for patients with 4–5 doses compared to those with fewer than 4 doses or no dose was 0.23 (95 % Confidence Interval: 0.11–0.44), while the vaccine effectiveness was 77 % and coverage 50.5 %. Genetic analysis of *B. pertussis* isolates from different Peruvian regions detected two clonal groups as identified by PFGE. Those two groups corresponded to the *B. pertussis* genotypes emerging worldwide *ptxP*3-*ptxA*1-*prn*2 or 9-*fim3-1* and *ptxP*3-*ptxA*1-*prn*2 or 9-*fim*3-2.

**Conclusions:**

Two emerging *B. pertussis* genotypes similar to isolates involved in worldwide epidemics were detected in Perú. Low vaccine coverage (<50 %) and genetic divergence between the vaccine-producing strain and the local isolates could contribute to this pertussal epidemic.

## Background

Whooping cough or pertussis is an acute respiratory disease caused mainly by the Gram-negative bacterium *Bordetella pertussis.* Despite the introduction of massive vaccination campaigns against pertussis in the fifties, the disease remains a relatively common infection with increasing incident rates reported in many countries [1–4]. Thus, pertussis is considered at present a serious public health problem in many countries, even including those with high vaccination coverage [2, 5–7]. According to the World-Health Organization (WHO), the incidence rates of the disease have increased, reaching about 16 million cases per year in the world and involving approximately 200,000 deaths, with 95 % of those cases occurring in developing countries [1].

Several causes have been proposed to explain the resurgence of pertussis, with most being associated with current vaccines: *e. g.*, a waning vaccine-induced immunity, the switch from whole-cell vaccines (wP) to acellular vaccines (aP), and an adaptation of the pathogen [8]. Published data on the duration of immunity have estimated that the immune protection after vaccination wanes after 4–12 years [9]. Recently, a *meta*-analysis of the duration of protective immunity to pertussis after a routine childhood immunization with aP indicated that the average duration of protection from the aP vaccine is about 3 years, assuming an 85 % vaccine efficacy [10]. The switch from the wP to the aP vaccine in many countries for the schedule of primary vaccinations resulting from concerns raised by reports of adverse reactions associated with wP has apparently complicated the pertussis epidemiologic situation [11, 12]. In that regard, a case-control clinical study designed to assess the risk of pertussis among adolescents and young adults during the 2010 outbreak in California, USA revealed that teenagers who had received four wP doses were nearly six times less likely to have been diagnosed with pertussis than those who had been given all aP vaccines and nearly four times less likely than those who had been given a mix of vaccines [13]. Waning immunity also occurs after wP vaccination as well as after an episode of pertussis [14].

Another possible cause of this present pertussis resurgence is the adaptation of *B. pertussis* to the immunity induced by the current vaccines [15]. In fact, genomic analyses of *B. pertussis* has revealed that a recent evolution of these bacteria has involved sweeps in which a novel vaccine antigen allele arises and largely replaces the previous dominant allele within that *B. pertussis* population. In some—but not all—countries, the emergence of allelic variants of the pertussis toxin–virulence-factor (*ptx*A and *ptx*P), the pertactin (*prn*), and the fimbriae (*fim*2 and *fim*3) genes matches with the disease resurgences occurring there. More recently, isolates that do not express one or more components of the acellular vaccines—and in particular the antigenic virulence factor pertactin—have emerged [16–19]. Increasing reports of pertactin-negative isolates now point to the possible occurrence of a positive selective pressure involving the suppression of pertactin expression, perhaps influenced by the switch from the whole cell vaccine to the acellular vaccine [20–23].

In Perú—a Latin American country that has used wP in the schedule for the primary series of vaccinations—the incidence of pertussis has increased from an average of 0.68 cases per 100,000 inhabitants in the 2007–2011 period, to 5.31 in 2012 [24]. In order to find a possible explanation for the epidemiologic resurgence of pertussis detected in Perú in 2012, we have analyzed pertussis epidemiologic clinical information, vaccine-related details, and genetic data from *B. pertussis*-specific genomic analyses.

## Methods

The surveillance of pertussis in Perú traditionally was based mainly on clinical suspicion. The confirmation of cases through laboratory results was limited because the only diagnostic method available except for cultivation was direct immunofluorescence until the introduction of analysis by the real-time polymerase-chain reaction (PCR) in 2012. The Instituto Nacional de Salud (INS, Perú) currently uses the real-time–PCR method to diagnose pertussis based on the detection of the specific DNA sequences IS481, IS1001, and IS1002 in order to recognize and distinguish between *B. pertussis* and *B. parapertussis* [25].

### Vaccination schedule

The current Peruvian vaccination program comprises three doses of the pentavalent vaccine (a combination vaccine containing the diphteric and tetanic toxoids, inactivated cells of *B. pertussis* bacteria, a conjugated polysaccharide of *Haemophilus influenzae* B type, and the surface antigen of Hepatitis-B virus) at 2, 4, and 6 months of age. In addition, the schedule includes two booster doses with the wP vaccine (DwPT: against diphtheria, pertussis, and tetanus) at 18 months and 4 years of age [26]. The pentavalent and DwPT vaccines currently used in Perú are from The Serum Institute of India.

### Vaccine efficacy

Patients over 18 months of age were classified into two groups: those who had completed the vaccination scheme with 4–5 doses, and those who had received fewer than 4 doses or no doses at all.

The attack rate [AR = (number of cases)/(popultion at risk) × 100] and the relative risk (RR, the ratio of the proportion of the treated cases to the proportion of the nontreated cases) of pertussis after 4–5 doses compared with the same parameter after fewer than 4 doses or no doses were calculated.

The attack rate [AR = (number of cases)/(popultion at risk) × 100] was first determined for both populations, and from those data the relative risk (RR = the ratio of the proportion of the treated cases to the proportion of the nontreated cases) was then calculated as the ratio of those two attack rates. Finally, the percent vaccine efficacy was computed by the following equation:

$$ \left(1{\textstyle \hbox{-}}\left[\mathrm{ARvaccinated}/\mathrm{ARunvaccinated}\right]\right)\times 100=\left(1{\textstyle \hbox{-} }RR\right)\times 100 $$

### Population

A cross-sectional study was performed from May through December 2012 in the Peruvian regions where pertussis had been previously reported—namely, Ayacucho, Ucayali, Lima, Huánuco, Cajamarca, Loreto, and Tacna [27]. The protocol of the study and informed consent-assent were approved by the Research Committee and the Institutional Ethics Committee from the INS.

Informed consent was signed by the parents or legal guardians of children under 7 years old, whereas informed assent was applied to children over 7 years old in addition to the informed consent of their parents or guardians. Samples were sent by the primary health-attention centers of the regions after previous coordination with their Regional Bureau of Health. The study involved 840 people suspected of pertussi who conformed to the inclusion criteria of the study.

#### Inclusion criteria

Patients of all ages hospitalized or receiving care with persistent or paroxysmal cough, or people who had been in contact with suspected cases of pertussis and signed informed consent were included.

#### Exclusion criteria

Patients who exhibited signs and symptoms attributable to chronic lung disease or a previously diagnosed respiratory allergy were excluded. The inclusion-exclusion criteria for the choice of research participants were blind to socioeconomic status, skin color, religion, sexual preference, place of birth, or pregnancy status.

Following the identification of a clinical case, a case-report form was completed with the information collected during the patient and physician interviews. After collection, specimens were routinely shipped to a laboratory for diagnosis. At the present time, the diagnosis is performed by real-time PCR (introduced in 2012) and bacterial cultivation.

### Sample collection

The nasopharyngeal samples were collected with polyester swabs having a flexible aluminum shaft (Puritan™, Fisher Scientific) following the World-Health-Organization Laboratory manual for pertussis [28] and the Pan-American Health Organization Guide [29, 30]. The swab samples were kept in vials with 500 μL of 0.9 % (w/v) sodium-chloride solution. Clinical samples were collected in hospitals and communities from Perú. Samples from patients suspected of pertussis or epidemiologically related contacts were sent together with their clinical and epidemiological records and informed-consent forms to the Laboratory of Biotechnology and Molecular Biology of the INS.

### Real-time PCR on clinical samples

Genomic DNA from clinical samples was extracted from 200 μL of nasopharyngeal swab supernatant and purified through the use of the Pure Link Genomic DNA Mini Kit (Invitrogen) according to the manufacturer's recommendations. The primers and probes used are listed in Table 1. The protocol for molecular diagnosis was performed as described previously [25]. For the PCR-reaction mixture, Platinum Quantitative PCR SuperMix-UDG (Invitrogen) was used. As a positive control for the real-time PCR assays, genomic DNA from the *B. pertussis* Tohama strain CIP 8132 was used.

**Table 1:** Primers and probes used in this study

Gene or sequence	Primer/probe sequence (5´–3´)^a^	Purpose	Reference
*IS481*	F: GCCGGATGAACACCCATAAG	Diagnosis	[25]
	R: GCGATCAATTGCTGGACCAT		
	P: (FAM)-CGATTGACCTTCCTACGTC-(BHQ1)		
*IS1001*	F: AATTGCTGCAAGCCAACCA	Diagnosis	[25]
	R: CCAGAGCCGTTTGAGTTCGT		
	P: (HEX)-ACATAGACCGTCAGCAG-(BHQ2)		
*IS1002*	F: CTAGGTCGAGCCCTTCTTGTTAAC	Diagnosis	[25]
	R: GCGGGCAAGCCACTTGTA		
	P:(CY5)CATCGTCCAGTTCTGTTGCATCACCC-(BHQ2)		
*ptx*P	F: AATCGTCCTGCTCAACCGCC	Genotyping	[33]
	R: GGTATACGGTGGCGGGAGGA		
*ptx*A	F: CCCCTGCCATGGTGTGATC	Genotyping	[33]
	R: TCAATTACCGGAGTTGGGCG		
*fim*2	F: GCGCCGGGCCCTGCATGCAC	Genotyping	[33]
	R: GGGGGGTTGGCGATTTCCAGTTCTC		
*fim*3	F: GACCTGATATTCTGATGCCG	Genotyping	[33]
	R: AAGGCTTGCCGGTTTTTTTTGG		
*Prn*	F: CAATGTCACGGTCCAA	Genotyping	[33]
	R: GCAAGGTGATCGACAGGG		

^a^ *F* forward primer, *R* reverse primer; *P* probes for IS481, IS1001, and IS1002. Fluorophores and quenchers are indicated at the 5´ and 3´ end of each probe, respectively

### *B. pertussis* isolation

Bacterial cultivation from the clinical samples was performed in Regan Low-Agar medium supplemented with cephalexin (40 μg/mL). The inoculated plates were incubated for 10 days at 37 °C under aerobic conditions at 95 % humidity. Bacterial colonies having features characteristic of *Bordetella* were confirmed as authentic by real-time PCR and then genotyped by genetic sequencing. A given culture was considered negative only after remaining colony-free for 10 days. Confirmed *B. pertussis* isolates were preserved in Brain-Heart Infusion broth containing 30 % (v/v) glycerol at −80 °C and recovered when needed for PFGE analysis.

### Pulsed-field gel electrophoresis (PFGE) of *B. pertussis* isolatess

PFGE analysis was performed according to references [31, 32] with some modifications. The procedure stated in brief: *B. pertussis* genomic DNA from 18 isolates in a Seakem Gold Agarose-gel plug was extracted from the bacteria with lysis buffer containing 1 % (w/v) sarcosyl and 100 μg/mL proteinase K and the plug incubated in a shaker bath at 56 °C for 16 h, and finally washed with 1X TE buffer six times at 50 °C in shaker bath. Genomic DNA was digested with the restriction enzyme *Xba*I at 37 °C for 6 h. Finally, electrophoresis was performed in a CHEF Mapper XA (Bio-Rad) apparatus for 23 h, with an initial pulse of 5 s and a final pulse of 35 s. The analysis and comparison of PFGE patterns were performed with Gel Compar II software, version 5.1 (Applied Maths), on the basis of the banding pattern of *Salmonella Branderup* (H9812) DNA as the normalization standard.

### Genotyping

DNA sequencing of *B. pertussis* genomic regions (*ptx*P, *ptx*A, *fim*2, *fim*, and *prn*) was performed with the primers listed in the Table 1, and according to the protocols of reference [33]. The PCR products in an agarose gel were purified with QIAquick Gel Extraction Kit (QIAGEN) and the sequencing reaction carried out with the BigDye Terminator v. 3.1 (Applied Biosystems) precipitated and resuspended in Hi-Di formamide (Life Technologies). The DNA sequencing was performed on an 3500XL Genetic Analyzer (Applied Biosystems).

DNA sequences were analyzed by means of the Sequencing Analysis and SeqScape Software (Applied Biosystems), with respect to the sequence assembly, alignments, and mutations. The sequences obtained were compared with reference sequences included in Genbank.

### Statistical analysis

We used the *Internet* Social Science Statistics Web Server (www.socscistatistics.com/tests/chisquare) for statistical comparisons between categorical variables by means of chi-square (*X*2) tests. P values of ≤0.05 were considered to indicate statistical significance.

## Results

### Perú pertussis outbreak in 2012

**Fig. 1:**
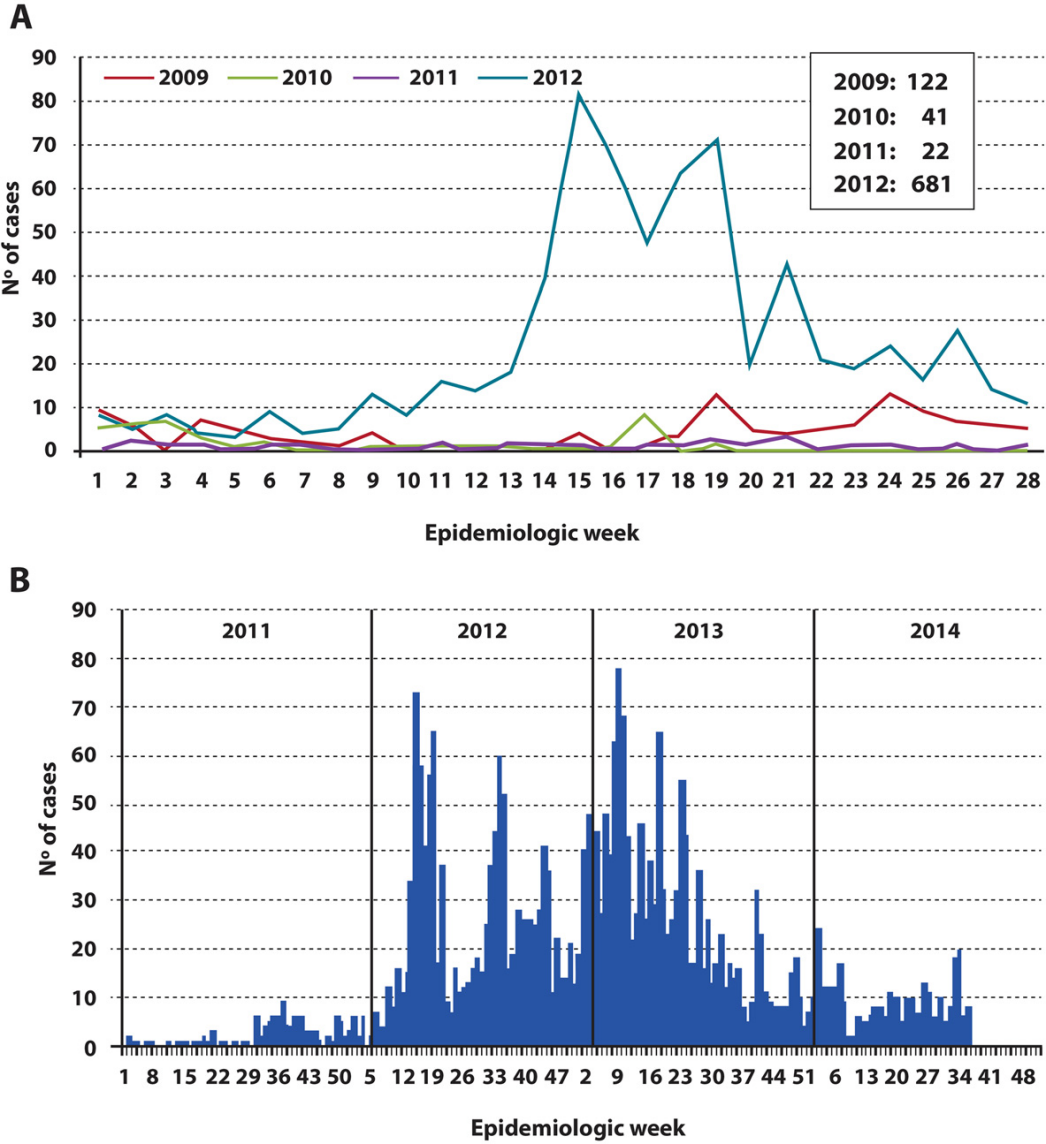
Weekly tally of pertussis-suspected cases according to year. Data obtained from the Peruvian Ministry of Health in 2012 (Panel **a** red curve, 2009; green curve, 2010; violet curve, 2011; azure curve, 2012) and 2014 (Panel **b** compressed curves for 2011 and 2012 next to the corresponding curves for 2013 and 2014 with the years indicated above the curves). In the figures, the number of cases is plotted on the *ordinate* as a function of the epidemiologic week on the *abscissa*

In 2012 an increase in pertussis cases was detected in comparison to the number of cases registered during the 2009–2011 period (Fig. 1, Panel a). Throughout 2013, the number of cases still remained higher than expected, but then decreased in 2014 (Fig. 1, Panel b) [34].

Of the total of 840 pertussis-suspected cases reported in 2012 in Perú (specifically in Ucayali, Ayacucho, Loreto, Lima Tacna, Cajamarca, Huánuco, Huancavelica, Piura), 390 (46.4 %) were male and 450 (53.6 %) female. The age of the patients ranged from a few days after birth to 83 years.

**Fig. 2:**
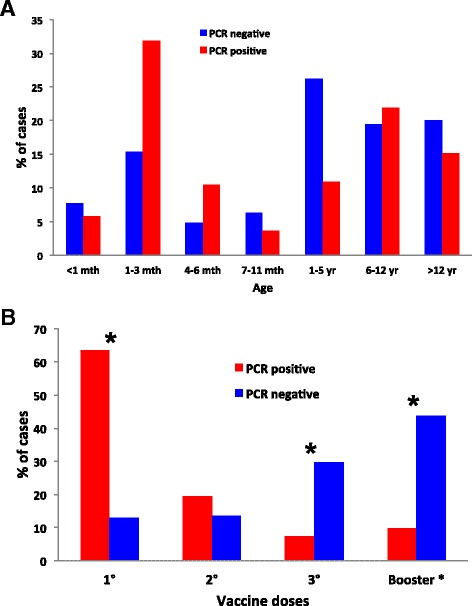
Distribution of pertussis cases detected in Perú during 2012 according to age (Panel **a**) or vaccination status of the patients (Panel **b**). In the figures, the percentage of the total number of suspected cases that were PCR-negative (blue bars) and PCR-positive (red bars) is plotted on the *ordinates* for each of the age ranges (Panel **a**) and for each of the vaccination doses (Panel **b**) indicated on the *abscissas*. The first three doses correspond to the pentavalent vaccine and the booster dose to the DTwP vaccine. The asterisk (*) indicates a significant difference in the chi-square test (*p* <0.00001)

The vaccination status was available for 588 of the patients: 464 (78.9 %) had had 0–3 pentavalent vaccine doses, and 124 (21.1 %) had received the primary scheme plus 1 or 2 booster doses performed with the DTwP vaccine (a total of 4 to 5 doses). Of the 408 patients of 6 months of age, 206 had received at least 3 vaccine doses (coverage: 50.5 %). Of the entire 840 surveyed cases, 191 (22.7 %) were positive in the assay by real-time PCR, (Ct ≤35), while 19 were positive by both PCR and cultivation. Eighteen of the suspected bacterial cultures were classified as *B. pertussis* and one as *B. parapertussis* (Table 2). A higher pertussis incidence was detected in the Peruvian regions of Ucayali, Loreto, Ayacucho, and Lima, (of the 840 total cases 30.9, 22.5, 17.3, and 11.5 % respectively; Table 3). The majority of the pertussis cases (60.7 %) were reported from July through September 2012 (Table 3) and detected in patients with fewer than 10 years of age (78 %; Fig. 2, Panel a). Approximately 52 % of patients with pertussis had started antibiotic treatment before the sample collection. The antibiotics used for the treatment of those confirmed pertussis cases were: azithromycin (for 30 % of the total), erythromycin (25 %), penicillins (22 %), and others (23 %; Table 2).

**Table 2:** Characteristics of patients suspected of pertussis in Perú during 2012

	PCR-negative *n* = 649 (%)	PCR-positive *n* = 191 (%)	*P* value
Gender			
Male	300 (46.2)	90 (47.1)	0.827
Female	349 (53.8)	101 (52.9)	
Vaccine			
Yes	265 (40.8)	82 (42.9)	**0.81**
No	182 (28.0)	59 (30.9)	
No data	202 (31.1)	50 (26.2)	
Vaccine doses^a^			
0–3 doses	331 (74.0)	133 (94.3)	<0.00001*
4–5 doses	116 (26.0)	8 (5.7)	
Clinical symptoms			
Persistent cough	460 (70.9)	160 (83.8)	0.00037*
Paroxysmal cough	416 (64.1)	147 (77.0)	0.00089*
Vomiting after coughing	97 (14.9)	114 (59.7)	<0.00001*
Stridor	177 (27.3)	71 (37.2)	0.0084*
Apnea	147 (22.7)	59 (30.9)	0.02
Cough (days)			
1–6	132 (20.3)	43 (22.5)	0.51
7–12	93 (14.3)	46 (24.1)	0.0014*
13–15	70 (10.8)	39 (20.4)	0.0005*
> 20	68 (10.5)	12 (6.3)	0.082
No cough	286 (44.1)	51 (26.7)	
Hospitalization			
Yes	113 (17.4)	67 (35.1)	<0.00001*
No	363 (55.9)	81 (42.4)	
Not indicated	173 (26.7)	43 (22.5)	
Antibiotic^a^			
Yes	197 (41.0)	100 (52.4)	−
Azithromycin	29 (14.7)	30 (30.0)	−
Erythromycin	56 (28.4)	25 (25.0)	−
Penicillins	54 (27.4)	22 (22.0)	−
Others	58 (29.4)	23 (23.0)	−
Antibiotic (days of treatment)^a^			−
1–3d	71 (10.9)	31 (16.2)	−
4–6d	28 (4.3)	16 (8.4)	−
≥ 7d	17 (2.6)	14 (7.3)	−
Not indicated	81 (12.5)	39 (20.4)	−
Etiology			−
*B. pertussis*	0 (0)	145 (75.9)	−
*B. parapertussis*	0 (0)	2 (1.0)	−
*Bordetella* sp.	0 (0)	44 (23.0)	−

*Chi-square (*X* ^2^) test (*p* <0.05)

^a^Percentages were calculated only in the population known to have been vaccinated or only in the recipients of an antibiotic

Of the total of 840 pertussis-suspected cases reported in 2012 in Perú, only 3 were treated with Cotrimoxazol

**Table 3:** Temporal and geographical distribution of patients suspected of pertussis in Perú during 2012

	PCR-negative *n* = 649 (%)	PCR-positive *n* = 191 (%)
Monthly pattern		
May	29 (4.5)	6 (3.1)
June	43 (6.6)	25 (13.1)
July	129 (19.9)	45 (23.6)
August	139 (21.4)	37 (19.4)
September	139 (21.4)	34 (17.8)
October	129 (19.9)	18 (9.4)
November	38 (5.9)	25 (13.1)
December	3 (0.5)	1 (0.5)
Regional distribution		
Ucayali	220 (33.9)	59 (30.9)
Ayacucho	201 (31.0)	33 (17.3)
Loreto	81 (12.5)	43 (22.5)
Lima	36 (5.5)	22 (11.5)
Tacna	56 (8.6)	17 (8.9)
Cajamarca	43 (6.6)	8 (4.2)
Huánuco	10 (1.5)	5 (2.6)
Huancavelica	2 (0.3)	2 (1.0)
Piura	0 (0)	2 (1.0)

The percentage of individuals with symptoms was significantly higher in patients with a positive laboratory result than in those who tested negative: persistent cough, 84 vs. 71 %, *p* = 0.00037; paroxysmal cough, 77 vs. 64 %, *p* = 0.00089; vomiting after cough, 59 vs. 15 %, *p* <0.00001; stridor, 37 vs. 27 %, *p* = 0.0084; 7–12 days of cough, 24 vs. 14 %, *p* = 0.0014; 13–15 days of cough, 24 vs. 14 %, *p* = 0.0014 (Table 2). Conversely, no significant differences were found for other symptoms—such as apnea or cough duration of less than one week or more than two weeks—or for gender or vaccine status without considering the number of doses (Table 2).

With respect to the number of vaccine doses, the percentage of patients suspected of pertussis was significantly higher in the group that had had 0–3 than in those that had had 4–5 doses (94.3 vs. 5.7 %, *p* <0.00001; *cf*. Table 4).

**Table 4:** Distribution of pertussis cases and no-cases by age and vaccination in Perú, 2012

			N° of vaccine doses						
	Age	n (%)	0	1	2	3	4	5	ND^b^
Cases	0–<6 m	84 (10)	43	19	6	-	-	-	16
	6–<12 m	13 (1.55)	2	-	8	-	-	-	3
	1–<2 y	5 (0.6)	1	2	1	-	-	-	1
	2–<4 y	9 (1.07)	-	3	-	-	3	-	3
	4–<6 y	7 (0.83)	-	2	-	-	3	-	2
	6–< 10 y	31 (3.69)	4	10	1	4	2	-	10
	10–<15 y	18 (2.14)	1	10	-	2	-	-	5
	15–<20 y	6 (0.71)	3	2	-	-	-	-	1
	20–<30 y	8 (0.95)	1	2	-	-	-	-	5
	30–<40 y	6 (0.71)	3	2	-	-	-	-	1
	>40 y	4 (0.48)	1	-	-	-	-	-	3
Non-cases	0–<6 m	150 (17.86)	82	21	6	3			38
	6–<12 m	54 (6.43)	15	1	11	17	-	-	10
	1–<2 y	54 (6.43)	5	1	2	15	10	-	21
	2–<4 y	60 (7.14)	2	6	6	1	27	3	15
	4–<6 y	64 (7.62)	6	2	1	5	19	22	9
	6–< 10 y	91 (10.83)	11	1	3	23	25	4	24
	10–<15 y	48 (5.71)	14	1	6	12	2	-	13
	15–<20 y	13 (1.55)	3	-	-	-	1	-	9
	20–<30 y	49 (5.83)	15	-	-	2	1	-	31
	30–<40 y	36 (4.29)	16	-	-	1	1	-	18
	>40 y	23 (2.74)	10	-	-	-	1	-	12
	No data of age	7 (0.83)	3	1	-	-	-	-	3
	Total	840 (100)	241	86	51	85	95	29	253

^a^Pentavalent vaccine for 1–3 doses plus DTwP for 4–5 booster doses

^b^ *ND* no data available from vaccine records

When pertussis was analyzed only in the group with known vaccination records; 27.2, 8.4, 3.1, 4.2, and 0 % had received 1, 2, and 3 doses, and those 3 along with one or two vaccine boosters, respectively. Conversely, the number of patients with diagnostic results that were pertussis-negative increased with the number of vaccine doses received (12.8, 13.6, 29.8, and 43.7 %, respectively; *cf*. the blue bars of Fig. 2, Panel b). Table 4 shows the distribution of pertussis-confirmed cases and non-cases according to age and vaccination status. The majority of those confirmed cases were detected in individuals of fewer than 6 months of age as well those having had fewer than 3 doses of vaccine (Table 4). As expected, non-cases received more vaccine doses than those confirmed cases.

For patients of more than 18 months of age, the AR for pertussis per vaccination status proved to be 28 % in those with 0–3 doses of vaccine and 6.4 % in those with 4–5 doses. Thus, the RR for patients with 4–5 doses compared to those with fewer than 4 doses or no doses at all was 0.23 (95 % confidence interval: 0.11–0.44), and the vaccine effectiveness was 77 %.

### Genomic analysis of *B. pertussis* isolates distributed throughout Perú

**Fig. 3:**
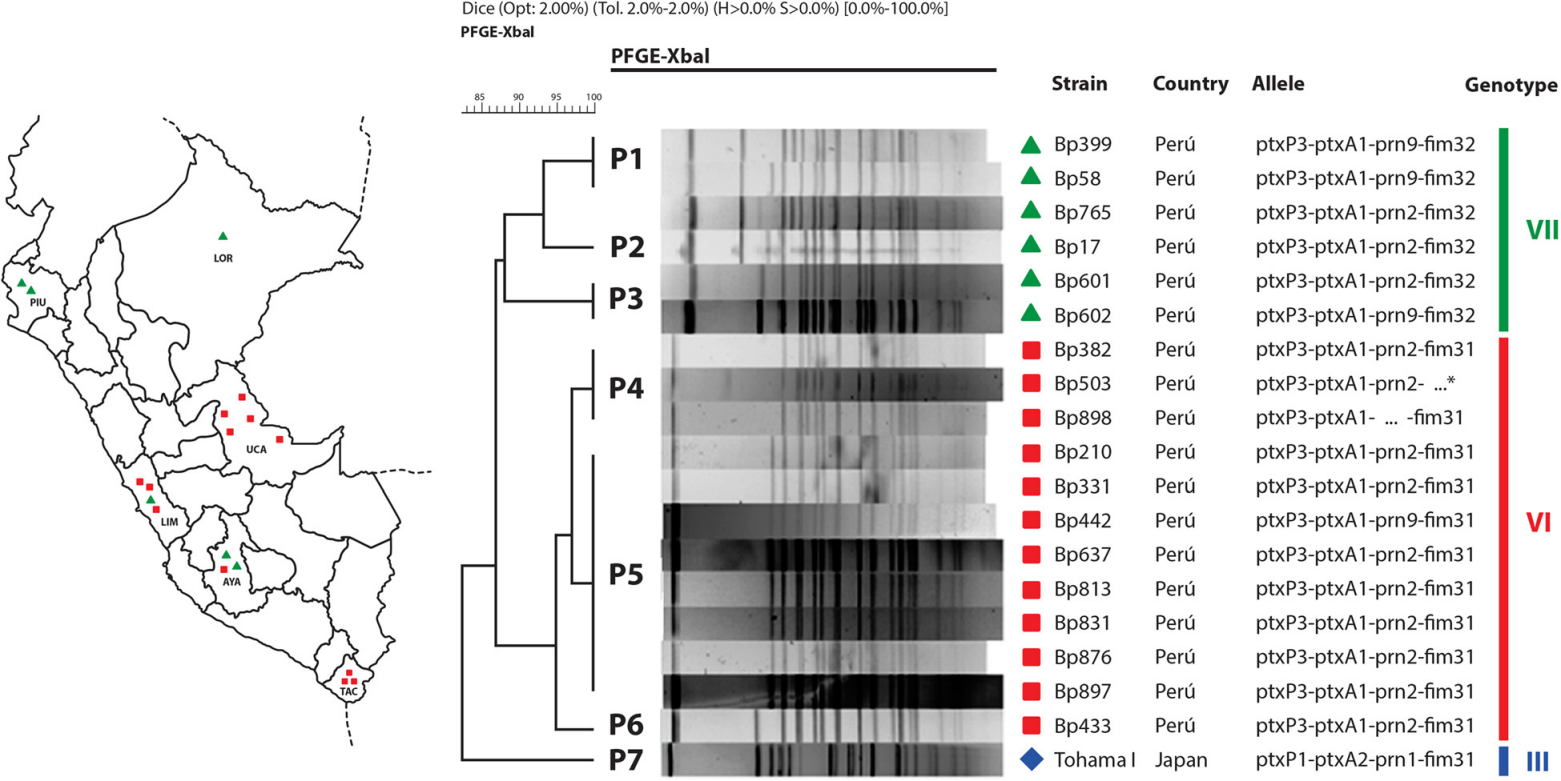
PFGE profiles and genotypes of *B. pertussis* Peruvian clinical isolates. Geographic distribution of *B. pertussis* isolates is shown on the left of the figure. The different individual patterns of pulse-field–gel-electrophoresis profiles in the center are indicated as P1 through P7. Genotyping was performed by sequencing the pertussis-toxin promoter (*ptxP*), pertussis-toxin subunit A (*ptxA*), pertactin (*prn*), *fimbriae 2* and *fimbriae 3*. The classification of the allelic genotypes (indicated as Bp-strain numbers to the left of the sequences along with the country of origin)—III (blue), VI (red group), and VII (green group)—is based on the van Gent et al. (2012) report [44]

*B. pertussis* clinical isolates (18) have been genetically characterized by PFGE and the DNA sequencing of the *ptx*P, *ptxA*, *prn*, *fim2*, *and fim3* genetic loci. Among the *B. pertussis* local isolates collected, 6 different PFGE-profile patterns were identified (Fig. 3, PFGE patterns P1–P6) and classified into 2 groups by means of the algorithm unweighted-pair-group method with averaging (UPGMA): the first group comprised the P1–P3 patterns while the second the P4–P6. These two major *B. pertussis* groups had a minimum of 87 % overall relatedness between them. *B. pertussis* Tohama strain (PFGE pattern P7), with less than 80 % similarity to that of the clinical isolates, was classified in a different PFGE group. The P1–P3 group consisted in more isolates than the P4–P6 (*i. e.*, 12 vs. 6), with P5 being the most highly represented PFGE pattern (with 8 isolates; Fig. 3).

A gene-sequencing analysis of virulent factors has categorized *B. pertussis* isolates (*n* = 18) into two genotypes: *ptxP*3-*ptxA*1-*prn*2 or 9-*fim*3-1, *fim*2-1 and *ptxP*3-*ptx*A1-*prn*2 or 9-*fim*3-2, *fim*2-1. Twelve and 5 isolates presented *prn*2 along with the less frequent *prn*9 genotype [35], respectively. As to the *fim*3 allele, 65 % was *fim*3-1 and 35 % *fim*3-2. We could not PCR-amplify either the *prn* gene sequence from a single isolate (Bp898), nor the the *fim*3 gene from another isolate (Bp503). As expected the *B. pertussis* Tohama phase-I–strain genotype was *ptxP*1-*ptxA*2-*prn*1-*fim*3-1.

## Discussion

During 2012 in Perú, an increase in pertussis cases was observed as reported by the National Surveillance System from the Peruvian Ministry of Health (Fig. 1) [34]. This increase could be attributable at least in part to the introduction of a specific real-time PCR as a laboratory diagnostic method instead of direct immunofluorescence along with polyester swabs instead of the previously used calcium-alginate swabs; which material, unlike polyester, is known to inhibit the PCR reaction [36, 37]. Moreover, the culture of *B. pertussis* was also reintroduced for use in the diagnosis of pertussis in that same year. The outbreak of pertussis detected at that time, could have also been influenced by the contemporary pertussis epidemics reported in neighboring countries [4, 30, 38]; which episodes in some instances had started before 2012, and whose detection had also been influenced by improvements in surveillance and laboratory diagnosis [4, 30, 38].

The increase observed in the pertussis incidence in 2012 in this country was sustained in 2013, but then decreased in 2014 (Fig. 1, Panel b), probably as a result of the reinforcement in pertussis immunization introduced after 2012.

During 2012 most of the pertussis cases were confirmed in patients younger than 3 months old (*p* <0.00001; Fig. 2, Panel a). A large proportion of cases recorded in patients younger than 3 months old was not unexpected since pertussis is most severe in that age group. Notwithstanding, the number of cases registered in other age groups should not be disregarded, especially during a pertussis epidemic in the country. As expected, young unvaccinated infants, or infants with an insufficient number of doses to confer adequate immunization, were more likely to contract a severer disease, suffer complications, and require hospitalization than older children and adults.

When patients with a positive diagnosis of pertussis were grouped according to the number of vaccine doses, the disease incidence in individuals who had received fewer than 4 (the pentavalent wP alone or no vaccine) was significantly higher than in those who had received 4–5 doses (pentavalent wP plus DTwP boosters; 94.3 vs. 5.7 %, *p* <0.00001; Table 2).

Another informative detail that became clear from the analysis was the distribution of patients with pertussis-positive diagnoses in relation to the age and vaccination status of the patient (Table 4, Fig. 2). Of the total number of studied cases, 69.4 % (583) had complete data regarding the patient's age and vaccination status, while 21 % (177) were younger than 6 months of age and had received fewer than 3 vaccine doses. Approximately 11.9 % (100) of the pertussis patients had had 3 or 4 vaccine doses and were between 6 months and under 6 years of age, whereas 3.45 % (29) had received 5 doses of vaccine (Table 4).

Pertussis-vaccination coverage in some regions of Perú was inadequate, and particularly with respect the first dose of booster (*ca*. 50 %). The influence of vaccination status on the incidence of pertussis was clearly demonstrated in this study since a statistically higher pertussis-attack rate was detected in individuals with 0–3 vaccine doses at 28 vs. 6.4 % in individuals with 4–5 vaccine doses (Fig. 2). These results once again underscore the need for improving vaccination coverage in all regions of Perú, in particular in those areas where poverty is prevalent.

As to the *B. pertussis* genotypes, a total of 6 different PFGE profiles, referred to as P1 through P6 were characterized in the clinical isolates. These profiles were classified into two major groups based on the criterion of a degree of similarity higher than 0.70. Genetic differences between bacteria detected and characterized throughout the country and the *B. pertussis* Tohama-phase-I strain were also observed (Fig. 3, sequences on the right). This result is expected since that strain is an older laboratory-adapted strain [39].

The predominant allelic variants that caused the Peruvian epidemic were: *ptxP*3-*ptxA*1-*prn*2 or 9-*fim*3-1 and *ptxP*3-*ptxA*1-*prn*2 or 9-*fim*3-2 (Fig. 3), with *ptxP*3-*ptxA*1-*prn*2-*fim*3-1 being the more prevalent genotype—it having been designated by other authors as MT27a [40]. The results detected for *fim* alleles were similar to those reported for the Argentine isolates *fim*2-1 (97 % of those studied), and *fim3-2* (76 %) [33].

The finding of the *prn*9 allele in 5 of the 18 total Peruvian *B. pertussis* isolates was unexpected in view of the low frequency previously reported for this allele [35]. *Prn2* is the most frequent allele present worldwide [15]. Nevertheless, the existence of isolates with the allele *prn*9 instead of *prn*2 or *prn*3 were quite prevalent, while the variant *ptx*P3 corresponded to the *ptxP* allele (Fig. 3). That the presence of *ptxP3* in the isolates was associated with an increased capacity to spread and with an enhanced mortality rate is both relevant and notable [41]. Nevertheless, that the complete genetic background of *ptxP*3 strains in addition to the *ptxP*3 must be likewise responsible for the better fitness of *B. pertussis* is also noteworthy [42, 43].

Unfortunately we could not obtain the *prn* genetic sequence for the isolate designated Bp898 (Fig. 3) probably because of mutations in the PCR-primer matching sequence or a deletion of the *prn* gene.

We must also stress that the *B. pertussis* isolates comprising group VI in Fig. 3 possessed a higher degree of genetic similarity than those of group VII. This classification of the allelic genotypes is based on the van Gent et al. (2012) report [44]. Moreover, many isolates collected in different regions of Perú presented the same P5 PFGE pattern. This correspondence suggests a clonal distribution of relatively few isolates throughout the entire country. These findings are in accordance with previous reports that revealed an increasing prevalence of the *B. pertussis* genotype VI in the last decade [44].

## Conclusions

Our results describe the pertussis epidemiologic situation in Perú and underscore the significance and urgency of enhancing the vaccine coverage. The genotypes of local circulating bacteria could be in part responsible for the epidemic described in this country in 2012, but molecular-genetic studies like those reported here should be performed in order to continue to characterize the pertussis-disease scenario in Perú.

## Abbreviations

DwPT, Diphtheria, whole pertussis, and tetanus; *fim*, Fimbria gen; PCR, Polimerase Chain Reaction; PFGE, Pulsed-field gel electrophoresis; *prn*, Pertactin gene; Ptx, Pertussis toxin; *ptx*, Pertussis toxin gen; *ptx*A, Pertussis toxin subunit A gen.; *ptx*P, Pertussis toxin gen Promoter
